# *Notes from the Field:* Autism Spectrum Disorder Among Children with Laboratory Evidence of Prenatal Zika Virus Exposure — Puerto Rico, 2023

**DOI:** 10.15585/mmwr.mm7229a5

**Published:** 2023-07-21

**Authors:** Nicole M. Roth, Camille Delgado-López, Lisa D. Wiggins, Nancy Nieves Muñoz, Sarah B. Mulkey, Leishla Nieves-Ferrer, Kate R. Woodworth, Glorimar Meléndez Rosario, Mariam Marcano Huertas, Cynthia A. Moore, Van T. Tong, Suzanne M. Gilboa, Miguel Valencia-Prado

**Affiliations:** ^1^Division of Birth Defects and Infant Disorders, National Center on Birth Defects and Developmental Disabilities, CDC; ^2^Puerto Rico Department of Health; ^3^G2S Corporation, Shavano Park, Texas; ^4^Division of Human Development and Disability, National Center on Birth Defects and Developmental Disabilities, CDC; ^5^Children’s National Hospital, Washington, DC; ^6^Department of Neurology, George Washington University School of Medicine & Health Sciences, Washington, DC; ^7^Department of Pediatrics, George Washington University School of Medicine & Health Sciences, Washington, DC; ^8^Goldbelt Professional Services, LLC, Chesapeake, Virginia.

Infection during pregnancy with Zika virus, a mosquitoborne flavivirus, can cause birth defects and neurodevelopmental abnormalities (*1*). Autism spectrum disorder (ASD) is a neurodevelopmental disability characterized by social and communication impairment and restricted or repetitive patterns of behavior or interests (*2*); possible associations between antenatal exposure to a limited number of viruses and ASD have been observed (*2*). The U.S. Zika Pregnancy and Infant Registry (USZPIR)* monitors children born during January 1, 2016–March 31, 2018, to women with laboratory evidence of Zika virus infection during pregnancy. This report used data from USZPIR and the Puerto Rico Autism Registry^†^ to estimate the prevalence of ASD diagnoses among children with possible prenatal Zika virus exposure and to describe prenatal characteristics and other outcomes by ASD diagnosis status. This activity was reviewed by CDC and was conducted consistent with applicable federal law and CDC policy.^§^

## Investigation and Outcomes

In Puerto Rico, any child who fails a standardized autism-specific screening, regardless of Zika virus exposure, receives a standardized evaluation at Puerto Rico Children with Special Health Care Needs Pediatric Program and Autism Centers^¶^ to confirm an ASD diagnosis by Diagnostic and Statistical Manual for Mental Disorders, Fifth Edition** criteria. Those who meet ASD criteria are included in the Puerto Rico Autism Registry.

Among 3,122 children reported to USZPIR in Puerto Rico, 109 (3.5%) had received an ASD diagnosis (Table). When analysis was restricted to 1,968 (63.0%) children who received a social-emotional or ASD-specific screener^††^ at age ≥18 months, 105 (5.3%) received an ASD diagnosis. No statistically significant differences were identified in the proportions of children with differing evidence of Zika virus exposure,^§§^ maternal symptoms,^¶¶^ pregnancy trimester of exposure,*** or Zika-associated birth defects between those with and without an ASD diagnosis. A higher percentage of children with an ASD diagnosis were male compared with those without an ASD diagnosis.

**TABLE:** Prenatal characteristics and child outcomes among live-born infants with and without a diagnosis of autism spectrum disorder* — U.S. Zika Pregnancy and Infant Registry, Puerto Rico, 2023

Characteristic	All children reported to USZPIR N = 3,122				Children with ASQ:SE-2 or M-CHAT-R/F^†^ at age ≥18 mos reported to USZPIR n = 1,968			
	With ASD diagnosis		Without ASD diagnosis		With ASD diagnosis		Without ASD diagnosis	
	No.	% (95% CI)^§^	No.	% (95% CI)^§^	No.	% (95% CI)^§^	No.	% (95% CI)^§^
**Total (row %)**	**109**	**3.5 (2.9–4.2)**	**3,013**	**96.5 (93.1–100)**	**105**	**5.3 (4.4–6.5)**	**1,863**	**94.7 (90.4–99.1)**
**Laboratory evidence of Zika virus infection**								
Possible Zika virus infection^¶^	60	55.0 (42.0–70.9)	1,668	55.4 (52.7–58.1)	57	54.3 (41.1–70.3)	944	50.7 (47.5–54.0)
Positive Zika virus NAAT result**	49	45.0 (33.3–59.4)	1,345	44.6 (42.3–47.1)	48	45.7 (33.7–60.6)	919	49.3 (46.2–52.6)
**Maternal symptoms^††^**								
Signs and symptoms of Zika virus disease	44	40.4 (29.3–54.2)	1,275	42.3 (40.0–44.7)	44	41.9 (30.5–56.3)	863	46.3 (43.3–49.5)
No signs and symptoms of Zika virus disease	65	59.6 (46.0–76.0)	1,738	57.7 (55.0–60.5)	61	58.1 (44.4–74.6)	1,000	53.7 (50.4–57.1)
**Trimester with first evidence of exposure^§§^**								
1st^¶¶^	45	41.3 (30.1–55.2)	1,102	36.6 (34.5–38.8)	44	41.9 (30.5–56.3)	685	36.8 (34.1–39.6)
2nd	42	38.5 (27.8–52.1)	1,129	37.5 (35.3–39.7)	40	38.1 (27.2–51.9)	711	38.2 (35.4–41.1)
3rd	22	20.2 (12.7–30.6)	782	26.0 (24.2–27.8)	21	20.0 (12.4–30.6)	467	25.1 (22.8–27.5)
**Child sex**								
Female	35	32.1 (22.4–44.7)	1,502	49.9 (47.4–52.4)	34	32.4 (22.4–45.3)	909	48.8 (45.7–52.1)
Male	74	67.9 (53.3–85.2)	1,511	50.1 (47.7–52.7)	71	67.6 (52.8–85.3)	954	51.2 (48.0–54.6)
Zika-associated birth defects***								
Yes	5	4.6 (1.5–10.7)	118	3.9 (3.2–4.7)	4	3.8 (1.0–9.8)	84	4.5 (3.6–5.6)
No	104	95.4 (78.0–100.0)	2,895	96.1 (92.6–99.7)	101	96.2 (78.4–100.0)	1,779	95.5 (91.1–100.0)
**ASD outcomes**								
**Family member with ASD diagnosis**								
Yes	33	30.3 (20.8–42.5)	—	—	31	29.5 (20.0–41.9)	—	—
No	58	53.2 (40.4–68.8)	—	—	56	53.3 (40.3–69.3)	—	—
Unknown	18	16.5 (9.8–26.1)	—	—	18	17.1 (10.2–27.1)	—	—
**Child’s age group when parent first noticed symptoms, mos**								
<6	6	5.5 (2.0–12.0)	—	—	6	5.7 (2.1–12.4)	—	—
6–12	27	24.8 (16.3–36.0)	—	—	26	24.8 (16.2–36.3)	—	—
13–18	31	28.4 (19.3–40.4)	—	—	29	27.6 (18.5–39.7)	—	—
19–24	19	17.4 (10.5–27.2)	—	—	18	17.1 (10.2–27.1)	—	—
25–30	5	4.6 (1.5–10.7)	—	—	5	4.8 (1.6–11.1)	—	—
31–36	10	9.2 (4.4–16.9)	—	—	10	9.5 (4.6–17.5)	—	—
37–42	3	2.8 (0.6–8.0)	—	—	3	2.9 (0.6–8.4)	—	—
43–48	3	2.8 (0.6–8.0)	—	—	3	2.9 (0.6–8.4)	—	—
49–54	2	1.8 (0.2–6.6)	—	—	2	1.9 (0.2–6.9)	—	—
Unknown	3	2.8 (0.6–8.0)	—	—	3	2.9 (0.6–8.4)	—	—
**Age group of ASD diagnosis, mos**								
Median (range)	39	(19.0–73.0)	—	—	39	(19.0–73.0)	—	—
18–25	17	15.6 (9.1–25.0)	—	—	17	16.2 (9.4–25.9)	—	—
26–33	24	22.0 (14.1–32.8)	—	—	22	21.0 (13.1–31.7)	—	—
34–41	21	19.3 (11.9–29.5)	—	—	21	20.0 (12.4–30.6)	—	—
42–49	15	13.8 (7.7–22.7)	—	—	15	14.3 (8.0–23.6)	—	—
50–57	13	11.9 (6.4–20.4)	—	—	12	11.4 (5.9–20.0)	—	—
58–65	17	15.6 (9.1–25.0)	—	—	16	15.2 (8.7–24.8)	—	—
66–73	2	1.8 (0.2–6.6)	—	—	2	1.9 (0.2–6.9)	—	—
**Level of support in social communication^†††^**								
Level 1	22	20.2 (12.7–30.6)	—	—	22	21.0 (13.1–31.7)	—	—
Level 2	47	43.1 (31.7–57.3)	—	—	46	43.8 (32.1–58.4)	—	—
Level 3	40	36.7 (26.2–50.0)	—	—	37	35.2 (24.8–48.6)	—	—
**Level of support in restrictive, repetitive behaviors^†††^**								
Level 1	25	22.9 (14.8–33.9)	—	—	24	22.9 (14.7–34.0)	—	—
Level 2	64	58.7 (45.2–75)	—	—	62	59.0 (45.3–75.7)	—	—
Level 3	20	18.3 (11.2–28.3)	—	—	19	18.1 (10.9–28.3)	—	—

**Abbreviations:** ASD = autism spectrum disorder; ASQ: SE-2 = Ages and Stages Questionnaires: Social-Emotional, Second Edition; M-CHAT-R/F = Modified Checklist for Autism in Toddlers, Revised with Follow-Up; NAAT = nucleic acid amplification test; USZPIR = U.S. Zika Pregnancy and Infant Registry.

* ASD diagnosis by *Diagnostic and Statistical Manual for Mental Disorder, Fifth Edition* criteria. https://www.psychiatry.org/psychiatrists/practice/dsm

^†^ https://agesandstages.com/products-pricing/asqse-2/; https://mchatscreen.com/

^§^ CIs were calculated assuming a Poisson distribution.

^¶^ Includes maternal, placental, or infant laboratory evidence of possible Zika virus infection during pregnancy based on serologic evidence of a Zika virus infection, or serologic evidence of an unspecified flavivirus infection.

** Includes maternal, placental, or infant laboratory evidence of confirmed Zika virus infection during pregnancy based on presence of Zika virus RNA by a positive NAAT (e.g., reverse transcription–polymerase chain reaction).

^††^ Signs and symptoms included fever, arthralgia, conjunctivitis, rash, and other clinical signs or symptoms that are consistent with Zika virus disease.

^§§^ Symptom onset date or date of earliest laboratory evidence of Zika virus infection was used to calculate trimester of exposure.

^¶¶^ Zika virus infections that occurred during the periconceptual period, which is defined as 4 weeks before last menstrual period, are included in the first trimester of exposure.

*** Zika-associated birth defects include selected congenital brain anomalies (intracranial calcifications, cerebral or cortical atrophy, abnormal cortical gyral patterns, corpus callosum abnormalities, cerebellar abnormalities, porencephaly, hydranencephaly, or ventriculomegaly/hydrocephaly); selected congenital eye anomalies (microphthalmia or anophthalmia; coloboma; cataract; intraocular calcifications; chorioretinal anomalies involving the macula, excluding retinopathy of prematurity; and optic nerve atrophy, pallor, and other optic nerve abnormalities); and microcephaly at birth (birth head circumference below the third percentile for infant sex and gestational age based on INTERGROWTH-21st online percentile calculator unless infants meet criteria for possible measurement inaccuracy. http://intergrowth21.ndog.ox.ac.uk/

^†††^ Level 1: requires support; Level 2: requires substantial support; Level 3: requires very substantial support.

Among the 109 children with an ASD diagnosis, most required substantial or very substantial support in social communication (79.8%) and restricted, repetitive behaviors (77.0%). The median age at ASD diagnosis was 39 months (range = 19–73 months), and 33 (30.3%) children with an ASD diagnosis also had a family member with an ASD diagnosis.

## Preliminary Conclusions and Actions

This analysis found that among children with Zika virus exposure reported to USZPIR from Puerto Rico, the prevalence of ASD diagnosis ranged from 3.5% to 5.3%, depending on the denominator. Estimated 2018 prevalence of ASD in general population samples in the continental United States ranged from 1.3% to 4.6% among children aged 4 years (*3*) and from 2.3% to 4.5% among children aged 8 years (*4*). A systematic analysis found a prevalence of 723 autism cases per 100,000 population (<1.0%) in Latin America and the Caribbean in 2016 (*5*).

The findings in this report are subject to at least three limitations. First, follow-up to age 5 years is not yet complete, and ASD can be identified even later in childhood. Second, comparators of ASD prevalence in the general Puerto Rico population are not yet available. As of 2023, Puerto Rico is a participating site for the Autism and Developmental Disabilities Monitoring Network^†††^ to conduct ASD surveillance among children aged 4 and 8 years. Finally, delays in referral of children for evaluation because of the COVID-19 pandemic might have lowered the estimated prevalence of ASD.

Additional information is needed to determine whether an association between Zika virus infection in pregnancy and ASD in children exists. Among children with prenatal Zika exposure, screening was reported for only two thirds. ASD-specific screening is recommended for all children to identify concerns as early as possible and minimize delays in intervention.^§§§^

## Footnotes

*
https://www.cdc.gov/pregnancy/zika/research/registry.html

†
https://www.salud.pr.gov/CMS/242

§ 45 C.F.R. part 46.102(l)(2), 42 U.S.C. Sect. 241(d); 5 U.S.C. Sect. 552a.

¶
https://www.salud.pr.gov/CMS/77

**
https://www.psychiatry.org/psychiatrists/practice/dsm

†† https://agesandstages.com/products-pricing/asqse-2/; https://mchatscreen.com/

§§ Includes maternal, placental, or infant laboratory evidence of confirmed Zika virus infection during pregnancy based on presence of Zika virus RNA by a positive nucleic acid amplification test (e.g., reverse transcription–polymerase chain reaction), possible Zika virus infection during pregnancy based on presence of serologic evidence of a Zika virus infection, or serologic evidence of an unspecified flavivirus infection.

¶¶ Signs and symptoms included fever, arthralgia, conjunctivitis, rash, and other clinical signs or symptoms consistent with Zika virus disease.

*** Symptom onset date or date of earliest laboratory evidence of Zika virus infection was used to calculate trimester of exposure.

†††
https://www.cdc.gov/ncbddd/autism/addm-network-sites.html

§§§
https://publications.aap.org/pediatrics/article/145/1/e20193447/36917/Identification-Evaluation-and-Management-of
